# Caregiver Experiences with Dementia-Related Feeding/Eating Difficulties

**DOI:** 10.3390/healthcare12020133

**Published:** 2024-01-07

**Authors:** Shobha Sharma, Nur Atiqah A. Halim, Puspa Maniam

**Affiliations:** 1 Speech Sciences Programme, Faculty of Health Sciences, Universiti Kebangsaan Malaysia, Kuala Lumpur 50300, Malaysia; 2 Centre for Healthy Ageing and Wellness (H-Care), Faculty of Health Sciences, Universiti Kebangsaan Malaysia, Kuala Lumpur 50300, Malaysia; 3 Department of Otorhinolaryngology, Kuala Lumpur General Hospital, Kuala Lumpur 50586, Malaysia

**Keywords:** caregiver, experiences, dementia, feeding, eating

## Abstract

This cross-sectional study explores caregivers’ perceptions of feeding/eating difficulties in persons living with dementia, their support provisions, and the associated burdens. Cognitive decline, behavioral symptoms, and physical issues contribute to the deterioration of feeding/eating activities in people with dementia. Inadequate support during mealtimes has adverse consequences. This study includes 31 caregivers who completed an online questionnaire with three sections: sociodemographic information, feeding/eating problems and required support for individuals with dementia, and caregiver burden and distress. The questions on feeding and eating problems were adapted from the Appetite and Eating Habits Questionnaire (APEHQ). The results show that nearly all persons living with dementia had symptoms of feeding/eating problems, requiring caregiver support, ranging from verbal assistance to full physical assistance. The caregivers reported high distress, which was positively correlated with dementia severity. The findings emphasize the importance of raising caregiver awareness about dementia’s impact on eating behavior, identifying effective mealtime care strategies, meeting nutritional needs, and emphasizing personal self-care. This research provides insights for healthcare professionals to develop targeted interventions, alleviate caregiver burden, improve mealtime experiences, and ensure adequate nutrition for persons living with dementia.

## 1. Introduction

Dementia is a complex condition characterized by a range of symptoms that affect memory, cognition, and social functioning, thereby impacting individuals’ everyday activities and interactions. Globally, approximately 55 million people are affected by dementia, with over 60% residing in low- and middle-income countries [1]. In Malaysia, the prevalence of dementia among older adults aged 60 and above was reported to be 8.5%, according to the 2018 National Health and Morbidity Survey [2]. Dementia affects behavioral and cognitive functioning decline, resulting in symptoms like memory loss, difficulty communicating or finding words, difficulty in reasoning or problem-solving, and personality changes.

Feeding/eating difficulties are among the consequences experienced by persons living with dementia, with approximately 50% of them encountering challenges related to food intake within eight years of disease onset [3]. Diminished cognitive and physical abilities adversely affect the ability to eat and proficiency in using utensils, resulting in difficulties with eating and finishing a meal, as well as the potential consumption of non-edible objects [4]. Persons living with dementia then become dependent on others for eating due to their decline in judgment and behavior [5]. Constant assistance will also be required to prevent swallowing disorders where food and fluid can be aspirated into the respiratory tract and result in severe consequences of aspiration pneumonia [6]. Hence, caregivers assume a crucial role in effectively addressing the challenges encountered by persons living with dementia during mealtimes.

Caregivers are the primary people who hold the most responsibility in the care and decision making of any individual who is unable to care for themself safely and effectively. The management of feeding/eating difficulties relies on the active involvement of caregivers, who may encompass family members, friends, or nursing staff [7]. They assume critical roles in aiding with eating and drinking, as well as ensuring the provision of nutritionally suitable food that prioritizes both health and safety [8], and they must ensure that the persons living with dementia are provided with adequate food intake during mealtimes [9]. They bear the duty of crafting meals infused with aromatic elements, a practice that is acknowledged for its substantial impact on augmenting meal anticipation and stimulating appetite [10,11,12]. Furthermore, caregivers assume a pivotal role in overseeing alterations in dietary and fluid intake patterns to forestall notable weight fluctuations in persons living with dementia. They also endeavor to cultivate agreeable and gratifying meal experiences for persons living with dementia, encompassing the creation of a comfortable dining milieu [8,13]. This conducive setting serves as a cornerstone for nurturing the bond and rapport between caregivers and care recipients [14].

Researchers [15] found that caregivers often feel pressure and anxiety during mealtimes. The extensive time, effort, strategies, and patience required when assisting persons living with dementia via hand-feeding can also result in challenges and potential failures, and ultimately lead to prolonged mealtime periods [16,17]. Caregivers revealed that they had issues in carrying out the tasks of planning and preparing meals as well as feeding and making dietary decisions for persons living with dementia under their care [7]. The necessity for continuous supervision and physical support to prevent spillage during feeding also results in increased caregiving time during meal periods [18]. Caregivers often experience concerns regarding the management of eating and drinking difficulties [19], primarily due to apprehensions about inadequate food intake by persons living with dementia [20]. Caregivers, however, should recognize the importance of providing support that focuses on maximizing the remaining abilities of persons living with dementia rather than assuming all tasks themselves [10], as this can result in increased caregiver burden and distress.

Researchers have determined that the level of caregiver burden is impacted by various stressors, including the condition of the person living with dementia, the necessity to make decisions regarding dementia care [21], as well as issues related to family and work. Additionally, poor family functioning in terms of affective responsiveness, problem solving, and communication has been identified as a contributing factor [22]. Caregiving practices vary across cultures due to differences in societal norms and expectations. It is a demanding and complex task that often falls on the shoulders of family members, particularly in Asian societies, where filial piety and family caregiving traditions are deeply ingrained [23,24]. A study conducted among Asian American caregivers revealed that the senses of loyalty and commitment to parents or spouses contribute to a bigger risk of caregiver burden [21,22,23,24,25], which is characterized by unique challenges stemming from cultural values, social expectations, and limited support systems. A study from China reported that when administered the Caregiver Burden Inventory, caregivers of persons living with dementia exhibited a high level of caregiver burden [26].

Other studies [23,24,25] conducted across several Asian locations, including China, Hong Kong, South Korea, the Philippines, Singapore, Thailand, and Taiwan, found significant differences in caregiver activity and caregiver burden. Similarly, one Malaysian study reported that care receiver factors (severity of illness) were the ones that influenced the perceived burden among the caregivers of persons living with dementia rather than caregiver factors (such as gender and duration of giving or kinship) [27]. Caregivers may additionally encounter greater difficulties when individuals with cognitive impairment perceive the provided assistance as unhelpful [28].

The burden of caregiving in Asian families has significant implications for the caregivers’ mental health, physical well-being, and overall quality of life. Studies have consistently demonstrated higher levels of psychological distress, depression, anxiety, and caregiver burnout among Asian caregivers [29,30,31]. Moreover, caregivers often neglect their own health needs, leading to increased rates of chronic illnesses and compromised self-care [32,33]. Recognizing the distinct needs of Asian family caregivers is crucial in developing effective interventions and support services. Research has shown that these may include caregiver support groups, counselling services, respite care programs, and community-based initiatives [28,34]. Collaborative efforts involving healthcare professionals, policymakers, and community organizations are necessary to enhance awareness, reduce stigma, and improve access to resources for Asian family caregivers [30]. While Malaysia, a multicultural nation, lies within Asia, caregivers’ perceptions regarding the feeding/eating difficulties in persons living with dementia, the type of support provided, and the burden and distress when caregiving remain unclear. In the context of caregiving for individuals living with dementia, particularly in Asia, the close involvement and dedicated support provided by friends or nursing staff often lead to an intimate bond, blurring the lines between professional care and familial relationships, where these caregivers virtually assimilate into the family unit. For this reason, the term “caregiver” in this manuscript has included not only family but also friends and personal home nursing staff. This study therefore aims to shed light on the specific burden experienced by caregivers in Malaysian families, emphasizing the need for targeted interventions and support services to address their distinct needs.

## 2. Materials and Methods

This cross-sectional study aimed to investigate caregivers’ perceptions of feeding/eating difficulties in persons living with dementia, the type of support provided, and the associated burden and distress by using a questionnaire. The questionnaire was adapted and modified from the Appetite and Eating Habits Questionnaire (APEHQ) developed by Ikeda et al. [35], which assesses five domains of eating difficulties: swallowing problems, appetite changes, food preferences, eating habits, and other oral behaviors. As there was no established questionnaire specifically addressing the type of support required by persons living with dementia during feeding and eating, the formulation of several questions was based on previous research by Liu et al. [9]. Additionally, a section of the questionnaire focused on assessing caregiver burden and distress, drawing from Volicer’s [21] work. Consequently, the questionnaire comprised 25 items distributed across three sections: sociodemographic information (13 items), feeding/eating problems and support required by persons living with dementia (5 items), and caregiver-perceived burden and distress (7 items). The questionnaire employed a combination of multiple choice, open-ended, 5-scale Likert questions, and was available in both English and Malay language versions. Written permission was received from the original authors of the APEHQ to adapt the questionnaire. The questionnaire was first trialed with 3 participants from the Alzheimer’s Disease Foundation of Malaysia to test the protocol and the comprehensibility of the questions in the questionnaire. The questionnaire in this study was administered via the Google Forms platform to comply with national COVID-19 pandemic restrictions. The trial participants reported that the questionnaire was clear and easy to answer but requested that a section be provided for further comments or suggestions. This modification was made to the final version of the questionnaire to be used in the study.

This study involved 31 caregivers of persons living with dementia, selected through purposive sampling. Eligible participants were Malaysian citizens serving as caregivers for individuals clinically diagnosed with any form of dementia and receiving oral feeding. Caregivers of individuals with complex medical issues such as serious psychiatric illnesses (e.g., schizophrenia or major depression), significant neurologic antecedents (e.g., brain trauma, brain tumor, epilepsy, or inflammatory diseases), or individuals who were tube-fed were excluded from the study.

The collected data were analyzed using Statistical Package for Social Sciences (SPSS) version 25.0. Descriptive analysis was performed to summarize the variables, with categorical variables presented in numbers and percentages, while continuous variables were presented as means and standard deviations.

Ethical approval for this study was obtained from the Ethics Committee of Universiti Kebangsaan Malaysia (JEP-2022-04).

## 3. Results

### 3.1. Sociodemographics

The descriptive statistics for the caregiver demographics revealed that a total of 31 caregivers of persons living with dementia participated in this study, with 90.3% females (*n* = 28) and 9.7% males (*n* = 3). Most of the participants were Malay (83.9%), followed by Chinese (9.7%) and Indian/Punjabi (3.2%). Most participants also fell within the age group of 31–50 years. The caregivers were mostly children of the persons living with dementia (56.3%). Seventeen (54.8%) caregivers had provided care for persons living with dementia for a duration ranging from 1 to 5 years, while the remaining 45.3% comprised those who had provided care for more than 5 years. Most of the caregivers were married and lived together with the persons living with dementia (64.5%).

### 3.2. Demography of the Persons Living with Dementia

Among the 31 persons living with dementia, 41.9% fell within the age range of 70 to 79 years, while 35.5% were below 70 years old. The lowest occurrence was observed in the age group of 80 to 89 years, accounting for 22.6%. A diagnosis duration of less than 3 years had the highest frequency in this sample. In terms of dementia severity, the moderate stage was most prevalent (54.8%), followed by severe (35.5%) and mild (9.7%). The severity of an individual’s dementia was based on the formal diagnosis by their neurologist, as reported by the caregiver of the person living with dementia.

### 3.3. Support Provided by Caregivers during Feeding/Eating

On average, the persons living with dementia presented with 11 symptoms of feeding and eating problems, with a maximum of 34 symptoms observed in a single individual. The findings displayed in Table 1 demonstrate that a significant majority of the persons living with dementia necessitated some form of support or assistance during feeding and eating activities (90.3%). Among them, a considerable proportion required high support (46.4%). Four types of support or assistance were provided: verbal, visual, partially physical, and fully physical. Verbal support was the most frequently offered support during feeding and eating (39.1%), followed by full physical support (22.5%), visual support (21.0%), and partial physical support (17.4%). Regarding verbal support, the caregivers primarily engaged in reminding individuals to initiate, continue, conclude, or finalize their meals (38.9%). Full physical support involved caregivers aiding the persons living with dementia in tasks such as opening containers, pouring beverages, and arranging their meal plates (51.6%). Furthermore, the caregivers primarily employed visual support by pointing out utensils or food items, indicating where individuals could obtain their food and drinks (41.4%). Lastly, the most prevalent form of partial physical assistance provided was caregivers directly passing food, drinks, and utensils to the persons living with dementia (62.5%).

### 3.4. Feeding/Eating Challenges Faced by Caregivers of Persons Living with Dementia

Table 2 presents the manifestations of feeding/eating difficulties among persons living with dementia. The table encompasses eight domains of feeding/eating problems, highlighting the prevalence of symptoms within each domain. The findings indicate that persons living with dementia exhibited the highest number of symptoms related to appetite change (23.3%). Following closely, food preference issues constituted the second most common domain (17.3%), while eating habits ranked third (14.4%).

Within the appetite change domain, the symptom that was the most frequently reported by caregivers was the need to restrict food intake for persons living with dementia (20%). Subsequently, an increase in appetite was reported by 16.3% of the participants. Moving on to the food preference domain, a significant majority of persons living with dementia demonstrated a preference for sweet foods compared to their previous dietary habits (33.3%).

Regarding eating behaviors, a prominent symptom was the extended time taken to consume meals, affecting 44% of the sample. On average, persons living with dementia experience approximately 11 symptoms related to feeding/eating problems. Notably, the maximum number of symptoms observed in a single person living with dementia reached 34, highlighting the multifaceted nature of these challenges.

### 3.5. Caregivers’ Perceived Burden and Distress

As presented in Table 3, the majority of the caregivers experienced some form of burden or stress while providing care for persons living with dementia (54.8%), and an additional 32.2% expressed the possibility of experiencing caregiver burden and stress. Among those who reported feeling burdened, the caregivers predominantly experienced extremely high levels of burden and distress (51.6%), followed by high (25.8%), moderate (16.1%), and very low (6.5%) levels. The primary contributing factor to the caregivers’ burden and distress was the manifestation of behavioral and psychological symptoms in their care receivers (41.9%). The second most prevalent reason for the caregiver burden was the caregivers’ perceived lack of knowledge and resources regarding how to effectively care for persons living with dementia (21.0%). Additionally, 17.7% of caregivers reported feeling overwhelmed as their care receivers became excessively dependent on them while they concurrently balanced familial and work-related responsibilities.

Several participants provided additional reasons for their feelings of burden/distress:

“*Caring for someone is good, but it can be overwhelming. It’s hard to balance personal life.*”

(Participant 4, grand-daughter)

“*I have to do everything. When others try to do it is not right and I have to re-do. More stress like that.*”

(Participant 7, adult child)

“*My mother took good care of me last-time. Now my turn to take care but sometimes like I am not doing enough for her. Always fighting inside me.*”

(Participant 8, adult child)

“*I got health problems also but no time to take care myself. The dementia problem bigger than my problem.*”

(Participant 11, daughter-in-law)

### 3.6. Caregivers’ Coping Strategies

The coping strategies employed by the caregivers are presented in Table 4, shedding light on the various approaches utilized to manage the burden and stress associated with caring for the person living with dementia. A significant majority of the caregivers (77.4%) reported employing some form of coping strategies, while the other caregivers (22.6%) indicated not utilizing any coping strategies. While the most prevalent coping strategy involved seeking help from others in the caregiving process (61.2%), only about half of the caregivers resorted to taking temporary breaks from caregiving as a means of coping (51.6%). Only under half of the caregivers or 14/31 (45.2%) actively searched for caregiving resources on the Internet to address the burden and distress they experienced.

Several participants who reacted to open-ended questions about the support required by caregivers when tending to individuals with dementia unveiled various significant aspects/topics. The majority of the caregivers expressed a need for physical assistance, particularly in tasks such as lifting and moving the care receiver, performing daily activities for the person living with dementia, and handling household chores. Additionally, the caregivers emphasized the importance of mental and emotional support, including encouragement from others and having a friend with whom they could share their challenges. These forms of support were seen as crucial in reducing stress levels and cultivating patience while caring for a person living with dementia. Furthermore, the caregivers expressed a desire for more information and guidance on dementia, including approaches for managing expectations as the conditions of their care receivers progressively worsen in the future.

Almost all caregivers stated the use of coping strategies. While many of them chose from the list of strategies, several caregivers listed their strategy under the comments section:

“*Cry a lot. Stress go down a bit*”

(Participant 8, adult child);

“*Pray and think of God*”

(Participant 10, daughter-in-law);

“*Sing God songs softly*”

(Participant 18, adult child);

“*Read Quran*”

(Participant 27, adult child);

“*Say prayers and talk to God*”

(Participant 30, sibling);

“*Clean house and pray*”

(Participant 31, grand-daughter).

### 3.7. Severity of Dementia and the Level of Caregivers’ Perceived Burden/Distress

A statistically significant positive relationship was found between the severity of dementia and the level of burden and distress experienced by the caregivers, as evidenced by the correlation analysis (r(29) = 0.450, *p* = 0.011). The correlation coefficient value falls within the range of “0.40 to 0.59,” indicating a moderate relationship between the two variables. Therefore, the severity of dementia and the level of burden and distress experienced by caregivers demonstrate a moderate relationship (r(29) = 0.450, *p* = 0.011). This relationship signifies a meaningful association between the two variables, indicating that they tend to vary or occur together in a manner that is unlikely to be attributed solely to chance.

## 4. Discussion

In this study, we primarily explored the experiences of caregivers of persons living with dementia who were currently undergoing oral feeding. As the study was carried out during pandemic restrictions, the sample size was limited (*n* = 31) and was carried out online. The experiences and challenges were inquired using a questionnaire adapted from the Appetite and Eating Habits Questionnaire [35]. Notably, all participants with dementia exhibited some form of feeding/eating difficulties. The average number of problems reported was 11.19 (SD = 5.461), with individuals experiencing as many as 34 disturbances in feeding/eating. Among the various domains, appetite change emerged as the domain with the highest number of feeding/eating difficulties among persons living with dementia. Additionally, most eating problems in persons living with dementia pertained to taking a long time to eat, indicating challenges in eating behaviors.

The findings of this study revealed that nearly all persons living with dementia required some form of support or assistance during feeding or eating activities. Four types of support or assistance were identified, including verbal, visual, partial physical, and full physical support. Verbal support/assistance, such as helping individuals start, continue, stop, or complete their meals, was the most provided form of support. This reliance on verbal support can be attributed to the decline in cognitive function, which hampers purposeful movements and the recognition of sensory stimuli [20].

Over half of the participants reported experiencing some level of burden and distress while providing care for the persons living with dementia under their care and reported high levels of distress, which is consistent with the existing literature indicating that 80% of caregivers of persons living with dementia faced high stress levels, with half experiencing comorbid depression [8]. The primary source of stress for caregivers was the behavioral and psychological symptoms exhibited by persons living with dementia. Previous studies have identified behavioral problems, particularly aggression, as significant factors contributing to caregiver burden [36]. Caregiving for persons living with dementia often involves numerous responsibilities, including personal care, managing medications, and dealing with challenging behaviors. These demands can lead to high levels of stress among caregivers. A study conducted in Taiwan [23] found that caregivers of persons living with dementia experienced significantly higher levels of stress compared to non-caregivers. The stress experienced by caregivers can contribute to physical health problems such as hypertension and cardiovascular issues. While the findings of the current study were similar in terms of the reported stress, the questionnaire used did not sufficiently enquire about other factors that could have contributed to this.

Our study findings indicated a moderate relationship between the severity of dementia and the level of caregiver burden and distress experienced due to the responsibilities during mealtimes. This finding aligns with reference [37], which identified the severity of dementia as one of the risk factors for caregiver burden. In a recent study [36], it was reported that caregivers of persons living with dementia who have severe symptoms experience a higher burden. This is due to the limited cognitive functions, problematic behaviors, and poor health statuses of the care receivers.

Caring for a person living with dementia can also take a toll on caregivers’ mental health. An alarming 96.8% of caregivers in this study reported experiencing feelings of sadness, helplessness, and social isolation, contributing to the increased risk of mental health disorders. Similar research has consistently shown higher rates of depression and anxiety among Asian caregivers, which may be related to the societal expectations of filial piety. A study conducted in Singapore [38] reported that caregivers of persons living with dementia had a higher prevalence of depression compared to the general population.

While coping strategies, in general, include seeking support from others and taking breaks, the participants in this study also cited prayer as a source of comfort, strength, and solace. Faith and trust in religion, as well as engaging in prayer, can provide a sense of purpose and a feeling of being connected to something larger than oneself. This practice is especially important in times of stress and is a common practice in Asia. Prayer can provide a sense of emotional support and can be a way for caregivers to express their feelings, seek guidance, and find solace in their faith. Engaging in prayer can serve as a coping mechanism, helping caregivers find inner peace and a sense of calm amidst the challenges they face. An individual’s use of prayer as a potential complementary treatment for depression suggests that it is critical for mental and physical health treatment providers to be aware of the use of prayer as a coping resource [39] and could potentially explain why almost half of the participants in this study reported that they were not sure about their feelings of stress and burden in caregiving, with approximately 23% of them reporting moderate to low levels of stress. Indeed, prayer can offer a sense of purpose and meaning, reminding caregivers that their efforts are valued and that they are making a positive impact on their loved ones.

Many caregivers believe that support systems play a crucial role in mitigating the negative impact of caregiving on caregivers’ health. Access to formal support services and community resources can help alleviate caregiver burden and promote better health outcomes. Seventy percent (70%) of the participants reported that they did not have easy access to support systems (family or professionals) or did not know where to seek assistance. This is concerning, as a study conducted in Japan [40] emphasized the importance of support groups, respite care, and counselling in reducing caregiver stress and improving mental well-being.

A research study conducted with a small sample size of caregivers of persons living with dementia presented several inherent limitations. Undoubtedly, the findings may lack generalizability as the characteristics and experiences of the participants may not necessarily be representative of the larger population or of those living in different areas of the country. The reduced sample size also diminishes the study’s statistical power, making it more challenging to detect significant effects or relationships. Lastly, although the study included some qualitative components, not all participants provided additional comments, potentially leading to an incomplete understanding of caregivers’ experiences.

It is important to approach findings from studies with small sample sizes with caution, and further research with a larger sample size is recommended [41]. However, considering these limitations in the interpretation of results and recommendations, these findings still underscore the significant challenges faced by caregivers in providing support during feeding/eating activities for persons living with dementia, highlighting the need for targeted interventions and support systems to alleviate caregiver burden and distress. By recognizing these limitations, future researchers can approach their findings with a well-informed perspective, appreciating both the depth and breadth of insights gained from their smaller studies.

## 5. Conclusions

In conclusion, the manifestation of feeding/eating problems in individuals living with dementia not only poses significant challenges for the affected individuals but also creates an immense burden on their caregivers, especially in Asian families, due to the cultural expectations and the general lack of awareness about the disease. The multifaceted nature of these issues, ranging from physical difficulties in swallowing to behavioral changes impacting dietary habits, often requires dedicated attention and specialized care. Caregivers often navigate a complex landscape and constantly adapt strategies to ensure proper nutrition and hydration while managing the emotional toll of witnessing the person living with dementia struggle with these challenges. The persistent nature of feeding/eating problems in dementia not only affects the individual’s health but also adds to the caregiver’s stress, impacting their own well-being. Access to formal support services, community resources, and interventions such as support groups, respite care, counselling, and religion/faith clearly plays a vital role in alleviating caregiver burden, reducing stress, and promoting better mental well-being in Asian families. By providing greater support and resources, healthcare systems and communities can assist caregivers in their vital role, ultimately improving the overall quality of care provided to persons living with dementia. Addressing these challenges demands comprehensive support systems, education, and resources to alleviate the burden on caregivers and enhance the quality of life for both persons living with dementia and those who care for them.

## Figures and Tables

**Table 1:** Type of support provided during feeding and eating.

Type of Support Provided during Feeding and Eating	*n*	Percentage
Required support/assistance during feeding and eating		
Yes	28	90.3
No	3	9.7
Amount of support required during feeding and eating		
Very high	13	46.4
High	5	17.9
Moderate	2	7.1
Low	8	28.6
Type of support provided during feeding/eating		
Verbal support		
Remind the persons living with dementia to start, continue, stop, or complete the meal	21	38.9
Give encouragement	20	37.0
Remind the persons living with dementia to take the correct utensils or food	13	24.1
Visual support		
Point to the utensils or food to indicate how to pick up food and drinks	12	41.4
Model the eating activity correctly	9	31.0
Tap the table to show where to take/put down utensils or food	6	20.7
Other (place the food in front of persons living with dementia/on the table)	2	6.9
Partial physical support		
Hand over food/drinks/utensils to the hands of persons living with dementia	15	62.5
Full physical support		
Help to open containers, pour drinks or set up the plate	16	51.6
Feed or bring utensils to the mouth	15	48.4

**Table 2:** Feeding/eating challenges in each domain.

Domain	Feeding and Eating Challenge	*n*	Percentage
Initiating feeding	Refuses food or shows strong dislike toward food	10	58.8
	Refuses feeding assistance	6	35.3
	Violent reaction to feeding	1	5.8
Maintaining attention	The person living with dementia is easily distracted	18	50.0
	The person does not continue to eat after starting to eat	8	22.2
	Cannot sit still, rises from the chair, or leaves table	6	16.7
	Falls asleep while eating	2	5.6
	Other	2	5.6
Bringing food into the mouth	Unable to move food from a plate into the mouth	14	66.7
	Unable to keep the mouth closed while feeding	4	19.0
	Other	3	14.3
Swallowing problem	Taking a long time to swallow	17	37.8
	Coughing or choking when swallowing	9	20.0
	Difficulty in swallowing foods	6	13.3
	Difficulty in swallowing liquids	3	6.7
	Other	1	2.2
Appetite change	Caregiver needs to limit the food intake	16	19.8
	Increase in appetite	13	16.1
	Reporting hunger	12	14.8
	Seeking out food between meals/snacking	11	13.6
	Loss of appetite	11	13.6
	Requesting more food	11	13.6
	Overeating at mealtime	4	4.9
	Reporting being over-full	3	3.7
Food preference	Prefers sweet foods more than before	20	33.3
	Drinking more soft or sweet drinks	11	18.3
	Drinking more tea/coffee or water	10	16.7
	Hoarding foods	7	11.7
	Changes in taste preference	5	8.3
	Adding more seasoning to the food	3	5.0
	Other	3	5.0
	Developing other food fads	1	1.7
Eating habits	Taking a long time to eat	22	44.0
	Decline in table manners	12	24.0
	Wanting to eat at the same time every day	6	12.0
	Wanting to cook or eat the same food every day	5	10.0
	Tending to eat foods in the same order	3	6.0
	Other	2	4.0
Other eating behaviors	Tendency to snatch or grasp any food items	13	35.1
	Tendency to over-fill mouth	9	24.3
	Chewing or sucking without trying to eat	8	21.6
	Eating non-edible items (PICA)	6	16.2
	Episodes of vomiting	1	2.7

**Table 3:** Caregivers’ perceived burden and distress relating to the provision of mealtime assistance.

Caregivers’ Perceived Burden and Distress	*n*	Percentage
Feels burden/stress while caring for the person living with dementia during mealtimes		
Yes	17	54.8
Maybe	10	32.2
No	4	12.9
Level of perceived burden/distress when caring for the person living with dementia during mealtimes		
Extremely high	16	51.6
High	8	25.8
Moderate	5	16.1
Very low	2	6.5
Reasons of feeling burden/distress when caring for the person living with dementia during mealtimes		
My care receiver shows behavioral and psychologic symptoms	26	41.9
I feel like I lack knowledge and resources on how to care for persons living with dementia	13	21.0
My care receiver is too dependent on me	11	17.7
I hold various responsibilities with family and work	11	17.7
I feel sadness, helplessness, and social isolation	30	96.8
I am not sure where to seek help	22	70.9
Other	1	3.2

**Table 4:** Coping strategies used by caregivers.

Coping Strategies Used by Caregivers	*n*	Percentage
Uses coping strategies to overcome the burden or stress		
Yes	24	77.4
No	7	22.6
Strategies used to overcome the burden or stress		
Ask for help from other people in caregiving	19	61.2
Take a temporary break from caregiving	16	51.6
Search on the Internet for caregivers’ resources	14	45.2
Join a support group for caregivers	12	38.7
Staying organized (e.g., make lists and establish a daily routine)	12	38.7
Set boundaries to the obligations or tasks that exceed the capability	11	35.5
Seek out professional help (e.g., doctors, hospitals, etc.) on how to manage persons living with dementia or the eating/feeding problems they face	11	35.5
Others (e.g., gardening, spending time with the person living with dementia, praying, singing)	30	97.0

## Data Availability

The data are contained within the article.
